# A Retrospective Analysis of Three Focused Attention Meditation Techniques: Mantra, Breath, and External-Point Meditation

**DOI:** 10.7759/cureus.23589

**Published:** 2022-03-28

**Authors:** Kirti Sharma, A. Gabriella Wernicke, Husneara Rahman, Louis Potters, Gopesh Sharma, Bhupesh Parashar

**Affiliations:** 1 College of Humanities and Sciences, Virginia Commonwealth University, Gainesville, USA; 2 Department of Radiation Medicine, Zucker School of Medicine/Northwell Health, Lake Success, USA; 3 Department of Biostatistics, Zucker School of Medicine/Northwell Health, Lake Success, USA; 4 Graduate School of Education, University of Pennsylvania, Philadelphia, USA

**Keywords:** breath, mantra, focused attention meditation, meditation, muse, mindfulness

## Abstract

Objective

The goal of this study is to compare the effectiveness of three different meditation techniques (two internal focus techniques and one external focus technique) using a low-cost portable electroencephalography (EEG) device, namely, MUSE, for an objective comparison.

Methods

This is an IRB-approved retrospective study. All participants in the study were healthy adults. Each study participant (n = 34) was instructed to participate in three meditation sessions: mantra (internal), breath (internal), and external point. The MUSE brain-sensing headband (EEG) was used to document the “total time spent in the calm state” and the “total time spent in the calm or neutral state” (outcomes) in each three-minute session to conduct separate analyses for the meditation type. Separate generalized linear models (GLM) with unstructured covariance structures were used to examine the association between each outcome and the explanatory variable (meditation type). For all models, if there was a significant association between the outcome and the explanatory variable, pairwise comparisons were carried out using the Tukey-Kramer correction.

Results

The median time (in seconds) spent in the calm state while practicing mantra meditation was 131.5 (IQR: 94-168), while practicing breath meditation was 150 (IQR: 113-164), and while practicing external-point meditation was 100 (IQR: 62-126). Upon analysis, there was a significant association between the meditation type and the time spent in the calm state (p-value = 0.0006).

Conclusion

This is the first study comparing “internal” versus “external” meditation techniques using an objective measure. Our study shows the breath and mantra technique as superior to the external-point technique as regards time spent in the calm state. Additional research is needed using a combination of “EEG” and patient-reported surveys to compare various meditative practices. The findings from this study can help incorporate specific meditation practices in future mindfulness-based studies that are focused on healthcare settings and on impacting clinical outcomes, such as survival or disease outcomes.

## Introduction

Meditation [1] is a practice of focusing the mind with the aim to reduce anxiety, experience peace, reduce side effects from treatments of various benign and malignant diseases, and prevent various chronic psychosomatic diseases. One definition of meditation is “a family of self-regulation practices that focus on training attention and awareness to bring mental processes under greater voluntary control and thereby foster general mental well-being and development and/or specific capacities such as calm, clarity, and concentration” [2]. There are other definitions, although there is no one universally accepted definition [3,4].

The practice of meditation involves techniques that have been originally published and noted in ancient Asian texts, such as the Gheranda Samhita by Swami Niranjanananda Saraswati [5], Yoga Sutras of Patanjali by Swami Satchidananda [6], and Samadhi: The Highest State of Wisdom by Swami Rama [7,8]. Additionally, Matko and Sedlmeier [9] lumped various meditative techniques into categories that can be practiced depending on the practitioner’s preference, philosophical background, and religious/spiritual background. Such techniques include repeating syllables, body scan, sitting in silence, and observation of thoughts, emotions, and physical sensations. There have been limited neuroscientific comparative studies analyzing various meditative techniques and showing differential brain activity among the various techniques [9,10]. Most of the research on meditation has been among the following four types: focused attention (e.g., on breath or the body), open monitoring, loving-kindness/compassion meditation, and mantra meditation (e.g., transcendental meditation). Among various types of meditation techniques that are mentioned in the literature, a simple categorization can be whether a mediation technique is internal or external. An example of an external meditative technique is to focus with open eyes on an inanimate and still object placed at a visible distance for a certain length of time. An example of an internal meditative technique is closing the eyes and focusing the mind on the movement of breath. In addition to this categorization, another challenge is to assess the individual preference for the type of meditation. Traditional methods of studying the effectiveness of meditation are by evaluating patient-reported surveys or interviews [11].

MUSE (SCR_014418) is a low-cost commercially available electroencephalography (EEG) portable event-related brain potential (ERP) system that can output a numerical value of the ability of the mind to stay in a calm or neutral state. The MUSE device contains seven sensors that detect brain waves as one meditates. Brain waves are of various kinds and frequencies, including beta waves (generated by an active mind) with 15-40 cycles/second and alpha waves (9-14 cycles/second), which are generated during the rest state after an active one [12]. MUSE gives a numerical value (in seconds) representing the practitioner’s ability to be in the following states of mind: active (restless) state, neutral (not restless, but not focused) state, and calm (focused) state [12]. MUSE has also been used to predict stroke severity, monitor for dementia and chronic pain, detect drowsiness, perceive mental stress, study mental disorders, and evaluate visually induced motion sickness [12].

The goal of this project is to compare external versus internal meditative techniques utilizing MUSE for measuring participants’ ability to remain in a neutral or calm state. We hypothesize that both external versus internal meditation techniques will result in the equal ability of participants' minds to remain in a neutral or calm state.

## Materials and methods

**Figure 1:**
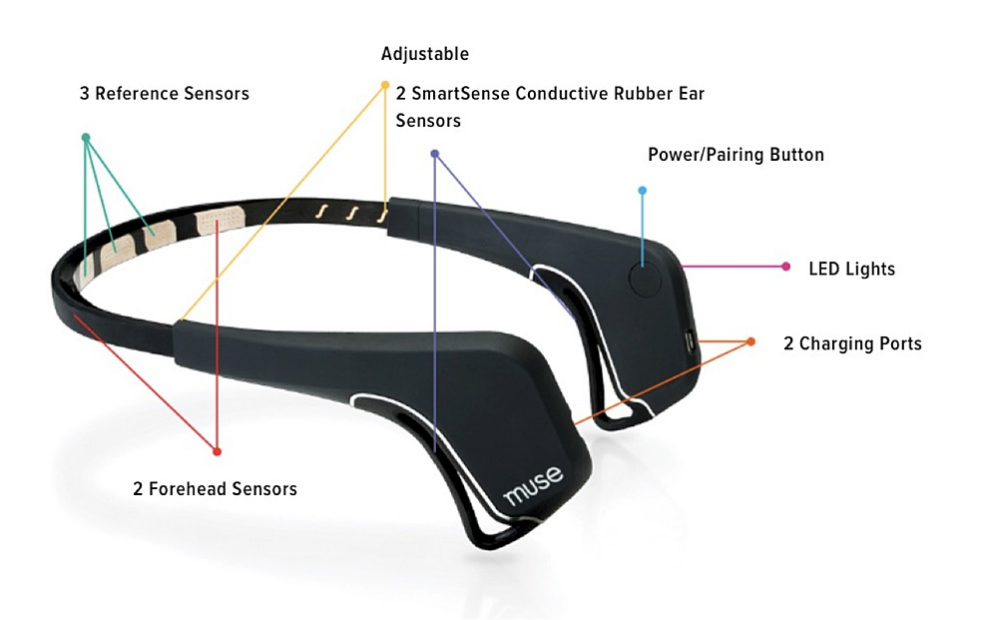
MUSE brain-sensing EEG headband

This retrospective study was approved by the IRB of Northwell Health. The aim of this study is to compare and determine the most effective meditation technique(s) that allow participants to achieve a neutral or calm state of mind. This data was collected on healthy participants, reporting no physical or mental ailments. The hypothesis was that all meditation techniques will produce similar effects on the mind. Data collection was done to compare the duration of neutral and calm points between three types of meditation using a brainwave-sensing headband, namely, MUSE. Figure 1 displays the MUSE device and its features (produced by Zhang [13]).

Healthy adult participants (n = 34) residing in the United States and India were included. The reason for including subjects from two countries was because the primary lead author volunteered at a monastery in India and lives in the United States and has access to such subjects. The decision about including a subject was made based on being an adult and healthy and having no obvious or reported mental issues. The demographic form consisted of the subjects’ age, gender, ethnicity, occupation, and experience in meditation. The level of each participant was based on whether a subject has “never” meditated (beginner), has recently started meditating in less than a year (intermediate), or has started meditating in more than a year (advanced). The subjects were all adults and “healthy” as reported by the subjects. Optional information included participants’ overall feedback of their experience in meditation and their preferred type. Participant demographics are shown in Table 1.

**Table 1:** Participant demographics

Participant characteristics	N (%)
Gender	
Male	12 (35.29)
Female	22 (64.71)
Age groups (years)	
18-28	5 (14.71)
29-38	8 (23.53)
39-48	10 (29.41)
49-58	7 (20.59)
59-68	4 (11.76)
Ethnicity	
Asian	22 (64.71)
Caucasian	12 (35.29)
Level of practice (participant reported)	
Beginner	13 (38.24)
Intermediate	14 (41.18)
Advanced	7 (30.59)

Each study participant was instructed to participate in three meditation sessions in the following order: mantra (internal), breath (internal), and external point. For the mantra and breath meditation, the participants were asked to close their eyes, while in the external-point meditation, their eyes were kept open. Each meditation session was timed through the MUSE’s mobile application for three minutes, with a combined time of nine minutes of meditation for each adult participant. Three minutes was chosen because of the ease of continuing meditation for a short time uniformly irrespective of whether a subject was new to meditation or more experienced. Permission was granted by each participant to help adjust the MUSE headband across the forehead and behind the ears for accurate calibration. To ensure that the headband was placed appropriately, the MUSE application displayed the functioning of the EEG sensors on each part of the band. Following the headband’s calibration, the participants were guided and directed to perform the meditation sessions.

For the mantra meditation, each adult was shown a brief list of universal mantras (a selection of Sanskrit words that imply peace, e.g., Om Namah Shivaya) to choose from for internal repetition. The subject was not required to disclose their chosen mantra if they did not feel comfortable sharing. In the second session, the participants were guided to perform the breath meditation and instructed to maintain focus on the natural flow of their breath. The first two sessions (mantra and breath) were performed with closed eyes. During the third session for the external-point meditation, the participants were asked to open their eyes without changing their seated position and then choose an object or point of focus in front of them. After the participant revealed their preference of focus, the researcher would make sure to approve or provide an alternative suggestion before moving forward. Any possible distractions within the participant’s peripheral view were also removed or managed before beginning the external-point meditation.

The “total time spent in the calm state” and “total time spent in the calm or neutral state” in the three-minute sessions were recorded and analyzed separately to compare the performance of the meditation techniques. The privacy of data was maintained throughout the study, and there was no disclosure of the participants’ specific research data to anyone outside the research study. Within the experimental analysis, there is no subject-identifying information, and all subjects are based outside the health system.

The summary statistics are given as median (IQR) for continuous data and frequency (percentage) for categorical data. The outcomes are also presented using boxplots of the raw data. Separate generalized linear models (GLM) with unstructured covariance structure were used to examine the association between each outcome (time spent in the calm state and time spent in the calm or neutral state) and the explanatory variable (meditation type). Associations between each demographic factor (age, gender, ethnicity, and level of practice) and each outcome were also examined. Because of the small sample size, it was not feasible to examine multivariable models. Therefore, univariate regression models were fitted using one explanatory variable at a time. For all models examining the time spent in the calm state, negative binomial regression was used. For all models examining time spent in the calm or neutral state, GLM with normality assumption was used. Due to the exploratory nature of the study, no adjustments for multiple testing were made, and a p-value < 0.05 was considered statistically significant unless otherwise noted. For all models, if there was a significant association between the outcome and the explanatory variable, pairwise comparisons were carried out using the Tukey-Kramer correction. All data were analyzed using the statistical software SAS version 9.4 (SAS Institute, Inc., NC, USA).

## Results

Thirty-four subjects were included in the study, including 22 females (65%) and 12 males (35%); 22 identified themselves as Asian, and 12 identified themselves as White. The age range was 18-68 years, although 74% of the patients were between 29 and 58 years of age. All 34 participants reported themselves to be generally healthy without any major medical issues. Of the participants, 79% identified themselves as “beginners” or “intermediate” level regarding meditation practice (Table 1).

Time spent in the calm state

**Figure 2:**
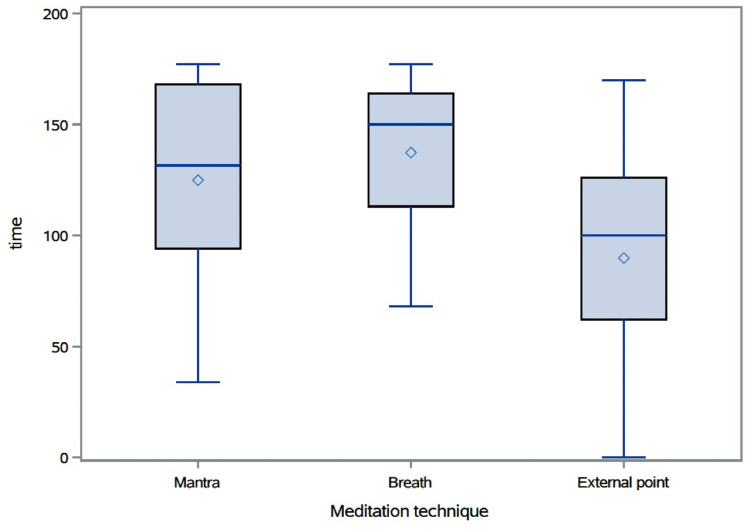
Time spent (seconds) in the calm state by meditation type

The median (IQR) time spent (in seconds) in the calm state while practicing mantra was 131.5 (IQR: 94-168), while practicing breath was 150 (IQR: 113-164), and while practicing external-point meditation was 100 (IQR: 62-126) (Figure 2). There was a significant association between meditation type and time spent in the calm state (p-value = 0.0006). Pairwise comparisons found that on average, the total time spent in the calm state was significantly longer while practicing breath than when practicing external-point meditation (adjusted p-value = 0.0001). The total time spent in the calm state was significantly longer when practicing mantra than when practicing external-point meditation (adjusted p-value = 0.0005). There was no significant difference between time spent in the calm state while practicing breath and time spent in the calm state while practicing mantra. No significant associations were found between age group, gender, ethnicity, level of meditation practice, and time spent in the calm state.

Time spent in the calm or neutral state

**Figure 3:**
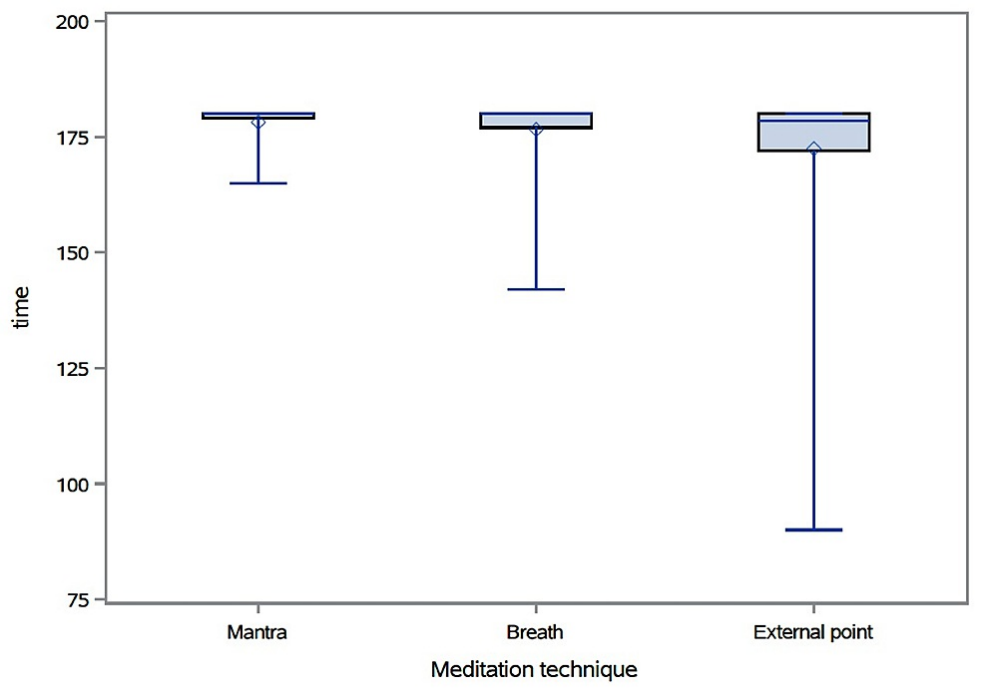
Time spent (seconds) in the calm or neutral state by meditation type

The median (IQR) time spent (in seconds) in the calm or neutral state while practicing mantra was 180 (IQR: 179-180), while practicing breath was 180 (IQR: 177-180), and while practicing external-point meditation was 178.5 (IQR: 172-180) (Figure 3). There was no significant association between meditation type, gender, or ethnicity and time spent in the calm or neutral state. There was a significant association between age group and time spent in the calm or neutral state (p-value = 0.037). We failed to detect which age groups differed significantly because of lack of power.

There was a significant association between the level of meditation practice and the duration of time spent in the calm or neutral state (p-value = 0.030) (Table 2).

**Table 2:** Time spent in the calm or neutral state and level of practice p-value = 0.030

Level of practice	Time spent in the neutral or calm state (mean in seconds)	Time spent in the neutral or calm state (lower level in seconds)	Time spent in the neutral or calm state (upper level in seconds)
Beginner	178.64	177.56	179.72
Intermediate	174.31	171.56	177.07
Advanced	172.95	164.37	181.54

Interestingly, beginners spent significantly more time in either the calm or neutral state than intermediate-level subjects (adjusted p-value = 0.012). There was no significant difference between the total time spent in the calm or neutral state by beginners and advanced participants. There was no significant difference between the time spent in the calm or neutral state by intermediate and advanced participants (adjusted p-value = 0.95).

## Discussion

As per the present study, both internal focusing techniques (mantra and breath) resulted in more calm or neutral points, suggesting a better focus compared with the external technique. The limitations of the study include its retrospective nature, small sample size, diverse demographic characteristics of subjects (although all were physically healthy), limited validation studies on the MUSE device, and variation in meditation experience among the participants. The present study adds to the growing literature on the best meditative and mindfulness-based practices that can be utilized for general well-being and in multiple medical situations.

Mindfulness meditation practice typically derives from various eastern traditions that involve both focused attention and open monitoring [14]. The practitioner continues to maintain and bring back focus in a nonjudgmental manner and later develops the skill of observing the mind functioning in a calm state. In open monitoring, the practitioner becomes aware of their thoughts, feelings, and sensations that may arise in the present moment.

Several published studies have compared meditative techniques. Amihai and Kozhevnikov compared the neurophysiological (EEG and EKG) and cognitive correlates of meditative practices that are thought to utilize either focused or distributed attention from both Theravada and Vajrayana (Buddhist) traditions [15]. The study showed that both focused and distributed attention meditations of the Theravada tradition produced enhanced parasympathetic activation, indicative of a relaxation response. In contrast, both focused and distributed meditations of the Vajrayana tradition produced sympathetic activation, indicative of arousal. Also, there was an immediate dramatic increase in performance on cognitive tasks following only Vajrayana styles of meditation, indicating enhanced phasic alertness due to arousal.

Lumma et al. compared three types of meditation, namely, breathing, loving-kindness, and observing-thoughts meditation, and assessed the heart rate (HR), high-frequency heart rate variability (HF-HRV), and subjective ratings of effort and likeability in their cognitive and attentional requirements [16]. HR and effort were higher during loving-kindness and observing-thoughts meditation compared with breathing meditation. With training over time, HR and likeability increased, while HF-HRV and the subjective experience of effort decreased. The increase in HR and decrease in HF-HRV after training was higher for loving-kindness meditation and observing-thoughts meditation compared with breathing meditation.

Fredrickson et al. studied day-to-day emotional profiles and dose-response relations, both within-persons and between-persons, associated with initiating one of two meditation practices, either mindfulness meditation or loving-kindness meditation [17]. Positive and negative emotions were also measured daily using the modified differential emotions scale. Multilevel models revealed significant dose-response relations between the duration of meditation practice and positive emotions, both within-persons and between-persons.

Burke conducted a pilot study comparing patient-reported preferences when analyzing four types of meditation: two open observing meditation techniques, Vipassana (mindfulness) and Zen, and two focused attention techniques, Mantra and Qigong visualization, practicing one method per week [18]. Significantly more participants chose Vipassana or mantra meditation as their preferred techniques compared with Zen and Qigong visualization.

In another comparative study, Qi et al. compared the psychological effects of meditation and breathing-focused yoga in a population of undergraduate students [19]. Four classes were randomized to meditation- or breathing-focused yoga. A total of 86 participants completed the surveys before and after the 12-week intervention, measuring work intention, mindfulness, and perceived stress. Students in the breathing-focused yoga group had significantly higher work intentions and mindfulness but marginally lower stress compared with the students in the meditation-focused yoga group.

Dib et al. conducted a medical intervention study comparing the effects of different relaxation interventions on physiological outcomes and perceived relaxation in healthy young women to identify the most appropriate intervention(s) for use in a subsequent trial for mothers who deliver prematurely [20]. The techniques were guided imagery meditation (GIM) audio, music listening (ML), relaxation lighting (RL), GIM + RL, and ML + RL, with control (silence/sitting) assigned in random order over a three- to six-week period. Based on preference, simplicity, and physiological and psychological effects, GIM and ML were identified as the most effective tools for reducing stress and improving relaxation.

Although we did not use the EEG part of MUSE for our study, the device is able to record brain waves in various meditative techniques [12]. There are several studies that have used EEG to evaluate brain activity during meditation [21-24]. A study evaluated the effect of Vipassana using EEG and found reduced delta waves and an increase in theta in the frontal regions of the brain [21]. There is possibly increased theta activity during Yoga Nidra and Sahaja Yoga meditation techniques [22,23]. In another study, strong delta and theta coherencies were seen during meditation with closed eyes, and no alpha coherency and changes in alpha and theta coherence during group meditation correlated with relaxation [24].

An interesting but unexplained finding in the present study is a better performance indicator of meditation practice in beginners compared with intermediate level practitioners for achieving a calm and neutral state of mind, as per the MUSE assessment. One possible explanation could be that more effort was made by the beginners in the study to remain in a calm or neutral state in the hope of performing well for the study. Another possible explanation is that beginners followed the researchers’ instructions better as compared with those who are at the intermediate or advanced level of meditation as they have their “pre-learned or preferred methods of doing a meditation.” Overall, the subjects did better in retaining a calm or neutral state when focusing “internally” rather than on an “external point.” This information can be useful for practitioners who are deciding between different meditation techniques when designing mindfulness interventions or courses.

## Conclusions

This is the first study comparing “internal” versus “external” meditation techniques using a low-cost portable MUSE device. Contrary to our hypothesis, based on the results of our study, “internal” meditation techniques result in a more “calm” state of mind. The current study has several limitations as discussed in the discussion section. Future studies should preferably be a prospective comparison and may use a combination of “EEG” and participant-reported surveys to compare various meditative practices.

## References

[1] Kral TR, Schuyler BS, Mumford JA, Rosenkranz MA, Lutz A, Davidson RJ (2018). Impact of short- and long-term mindfulness meditation training on amygdala reactivity to emotional stimuli. Neuroimage.

[2] Walsh R, Shapiro SL (2006). The meeting of meditative disciplines and Western psychology: a mutually enriching dialogue. Am Psychol.

[3] Deshmukh VD (2006). Neuroscience of meditation. ScientificWorldJournal.

[4] Jaseja H (2009). Definition of meditation: seeking a consensus. Med Hypotheses.

[5] Saraswati SN (2012). Gheranda Samhita/commentary on the yoga teachings of Maharshi Gheranda. Yoga Publications Trust/Munger/India.

[6] Satchidananda SS (2012). The yoga sutras of Patanjali. Integral Yoga Publications.

[7] Chowdhary S, Gopinath JK (2013). Clinical hypnosis and Patanjali yoga sutras. Indian J Psychiatry.

[8] Rama S (2002). The highest state of wisdom: yoga the sacred science. HIHT.

[9] Matko K, Sedlmeier P (2019). What is meditation? Proposing an empirically derived classification system. Front Psychol.

[10] Goyal M, Singh S, Sibinga EM (2014). Meditation programs for psychological stress and well-being: a systematic review and meta-analysis. JAMA Intern Med.

[11] Przyrembel M, Singer T (2018). Experiencing meditation - evidence for differential effects of three contemplative mental practices in micro-phenomenological interviews. Conscious Cogn.

[12] Krigolson OE, Williams CC, Norton A, Hassall CD, Colino FL (2017). Choosing MUSE: validation of a low-cost, portable EEG system for ERP research. Front Neurosci.

[13] Zhang R (2018). The effect of meditation on concentration level and cognitive performance. Glob J Health Sci.

[14] Lutz A, Slagter HA, Dunne JD, Davidson RJ (2008). Attention regulation and monitoring in meditation. Trends Cogn Sci.

[15] Amihai I, Kozhevnikov M (2014). Arousal vs. relaxation: a comparison of the neurophysiological and cognitive correlates of Vajrayana and Theravada meditative practices. PLoS One.

[16] Lumma AL, Kok BE, Singer T (2017). Corrigendum to "Is meditation always relaxing? Investigating heart rate, heart rate variability, experienced effort and likeability during training of three types of meditation" [Int. J. Psychophysiol. 97/1 (2015) 38-45]. Int J Psychophysiol.

[17] Fredrickson BL, Boulton AJ, Firestine AM (2017). Positive emotion correlates of meditation practice: a comparison of mindfulness meditation and loving-kindness meditation. Mindfulness (N Y).

[18] Burke A (2012). Comparing individual preferences for four meditation techniques: Zen, Vipassana (mindfulness), Qigong, and mantra. Explore (NY).

[19] Qi X, Tong J, Chen S, He Z, Zhu X (2020). Comparing the psychological effects of meditation- and breathing-focused yoga practice in undergraduate students. Front Psychol.

[20] Dib S, Wells JC, Fewtrell M (2020). A within-subject comparison of different relaxation therapies in eliciting physiological and psychological changes in young women. PeerJ.

[21] Chan AS, Han YM, Cheung MC (2008). Electroencephalographic (EEG) measurements of mindfulness-based Triarchic body-pathway relaxation technique: a pilot study. Appl Psychophysiol Biofeedback.

[22] Aftanas LI, Golocheikine SA (2002). Non-linear dynamic complexity of the human EEG during meditation. Neurosci Lett.

[23] Kjaer TW, Bertelsen C, Piccini P, Brooks D, Alving J, Lou HC (2002). Increased dopamine tone during meditation-induced change of consciousness. Brain Res Cogn Brain Res.

[24] Kora P, Meenakshi K, Swaraja K, Rajani A, Raju MS (2021). EEG based interpretation of human brain activity during yoga and meditation using machine learning: a systematic review. Complement Ther Clin Pract.

